# Two-Step Multilevel Latent Class Analysis in the Presence of Measurement Non-Equivalence

**DOI:** 10.1080/10705511.2025.2490946

**Published:** 2025-05-05

**Authors:** Johan Lyrvall, Jouni Kuha, Jennifer Oser

**Affiliations:** ^a^ University of Catania; ^b^ London School of Economics and Political Science; ^c^ Ben-Gurion University of the Negev

**Keywords:** Differential item functioning, latent variable models, random effects models, stepwise estimation, two-step estimation

## Abstract

We consider estimation of two-level latent class models for clustered data, when the measurement model for the observed measurement items includes non-equivalence of measurement with respect to some observed covariates. The parameters of interest are coefficients in structural models for the latent classes given covariates. We propose a two-step method of estimation. This extends previously proposed methods of two-step estimation for models without non-equivalence of measurement by specifying the model used in the first step in such a way that it correctly accounts for non-equivalence. The properties of these two-step estimators are examined using simulation studies and an applied example.

## 1. Introduction

The methodological research question that is considered in this article is the following: How can we estimate multilevel latent class models with covariates when there is non-equivalence of measurement in some of the measurement items, using the two-step method of estimation? How well do these estimates perform? We begin by briefly introducing the key terms in this statement.

*Latent class (LC) analysis* (Goodman, 1974; Lazarsfeld & Henry, 2004) is used to classify units into subgroups based on multiple observed categorical variables. The LC model takes these observed variables (*items*) to be indicators of a categorical latent variable of interest (*latent class*). For example, Oser et al. (2023) used LC analysis to identify types of citizenship norms measured by responses to multiple survey questions about different democratic values.

In applied LC analysis, substantive research questions commonly focus on associations between external predictors, or *covariates*, and the probabilities of belonging to the different latent classes. This is operationalised in terms of regression models for the classes given the covariates. For example, Oser et al. (2023) used socioeconomic predictors to describe how individuals sort into citizenship norms. The model then combines two elements: a *measurement* model for how the items measure the latent classes, and a *structural model* for how the latent classes depend on the covariates.

Basic LC modelling assumes that the units of analysis are independent of each other. This is insufficient when we have hierarchical data where *lower-level units* (such as individual respondents) are nested (clustered) within *higher-level units* (groups). The nesting can extend to still higher levels, but our discussion is limited to the case of two-level hierarchical data. It is assumed that units in different groups are independent of each other, but that lower-level units within the same group need not be independent even conditional on the covariates.

Within-group dependencies can be accommodated by introducing another latent variable which varies at the higher level. When it is categorical, i.e., a higher-level latent class variable, we have a *multilevel latent class model* (Vermunt, 2003). For example, Di Mari et al. (2023) used multilevel LC analysis to identify citizenship norms within countries, finding two country-level clusters with different prevalences of the individual-level classes of citizenship norms. The higher-level variable is analogous to continuous *random effects* in multilevel models which include such variables (see e.g., Rabe-Hesketh & Skrondal 2022 for examples of them). Multilevel LC models can include covariates as predictors of both higher- and lower-level latent classes. Most often substantive interest is focused on the lower level. For instance, Di Mari et al. (2023) identified socioeconomic predictors of individual-level norms.

We consider likelihood-based estimation of the models. In standard maximum likelihood (ML) estimation, or *one-step estimation*, all the parameters are estimated simultaneously. In contrast, *stepwise estimation* divides estimation of the measurement model and the structural model into separate steps. The one-step approach has the standard optimality properties of ML estimation, but it also has serious drawbacks (see the discussions in Vermunt, 2010 and Bakk & Kuha, 2018). Practically, it can be computationally demanding and will require the same computational effort every time the model is changed and re-fitted. Conceptually, estimating the measurement and structural models together has the disadvantage that they will affect each other. Any changes to the structural model, such as adding or removing covariates or changing their functional form, will also change the estimated measurement model, and hence the implied definition of the latent classes. These changes can be so large that they render comparisons of different structural models effectively meaningless.

Stepwise estimation avoids or reduces the disadvantages of the one-step method. It begins by estimating just the parameters of the measurement model (step 1). Different stepwise methods differ in what happens next. *Three-step estimation* assigns observations to the latent classes based on the estimated measurement model (step 2), and then fits the structural model for these assigned classes (step 3). *Bias-adjusted three-step estimation* employs further adjustments to correct for misclassication bias that would arise from naive use of step 2 (see the review in Bakk & Kuha, 2021 and references therein).

In contrast, stepwise *two-step estimation* does not assign predicted latent classes, but estimates (in its step 2) the structural model directly from a likelihood where the measurement-model parameters are fixed at their estimates from step 1. Two-step estimation for LC models was first proposed by Bandeen-Roche et al. (1997) and Xue and Bandeen-Roche (2002), and further developed by Bakk and Kuha (2018). The same idea can also be applied to latent variable models which have continuous rather than categorical latent variables (Kuha & Bakk 2023; Rosseel & Loh, 2024).

For multilevel LC models, stepwise methods have been proposed using a bias-adjusted three-step (Lyrvall et al., 2024), an intermediate “two-stage” (Bakk et al., 2022), and the two-step approaches (Di Mari et al., 2023). We regard the two-step method as the preferred approach because of its simplicity and good performance in previous studies.

A latent variable model has the property of *measurement equivalence* if the measurement model for the items depends *only* on the latent variables but not on any covariates or observed response variables. Violation of this, where measurement is affected also by observed external variables, is known as *measurement non-equivalence*, also known as non-invariance of measurement or differential item functioning (DIF). It can arise, for example, in cross-national surveys from differences in translation or in educational testing from differences in familiarity of test questions for different groups of students which are unrelated to their ability. In the illustrative example that we consider in Section 5 of this paper, we allow for possible non-equivalence in survey questions on citizenship norms which may arise from differences in the salience of different civic activities in countries with higher or lower levels of political freedom. There is a large literature on issues of non-equivalence in different applications and for different types of latent variable models (see e.g., Millsap 2011 and Kankaraš et al. 2011, and references therein). Masyn (2017) discusses it for LC models, and provides definitions and model specifications.

If there is non-equivalence in the measurement, estimation which ignores this will yield biased estimates also for the structual model. Studies by Asparouhov and Muthén (2014), Janssen et al. (2019) and Di Mari and Bakk (2018) show that this bias can be large for latent class models. It is thus often crucial to correctly account for any non-equivalence in model specification and estimation.

One-step estimation in this situation is still standard ML estimation, now for a model which includes covariates also in the measurement model. For stepwise methods, Vermunt and Magidson (2021) described how bias-adjusted three-step estimation can be implemented for single-level LC models with non-equivalence of measurement. Their key point is to specify the model for its step 1 correctly. This should include those covariates which affect the measurement model, and include them in both the measurement model and the structural model (they should then also be appropriately accounted for in steps 2 and 3).

Vermunt and Magidson (2021) also note that what they propose for three-step estimation would also be the correct form for step 1 of the two-step method. In this paper we follow up on that point. We combine the elements from previous literature described above, and extend them to develop two-step estimation which allows for non-equivalence of measurement and which can be applied to single-level and multilevel LC models.

The model is defined in Section 2 of the paper, and in Section 3 we describe how the estimation is implemented. We then evaluate the performance of the method through simulation studies in 4 and illustrate it further with an empirical example in Section 5.

## 2. Multilevel Latent Class Model with Covariates and Measurement Non-Equivalence

Here we give a formal definition of the model that was outlined in Section 1. We define its elements in steps, finishing with the introduction of non-equivalence to the measurement model.

Consider hierarchical data where *lower-level units* (individuals) $j=1,\ldots ,n_{i}$ are nested in *higher-level units* (groups) i=1,…,I. Let $Y_{\text{ijh}}\text{,}$ h=1,…,H, be the values of *H* observed variables (*items*) for lower-level unit *j* in higher-level unit *i*, and define $Y_{\text{ij}}={\left(Y_{\text{ij}1},\ldots ,Y_{\text{ijH}}\right)}^{\prime }\text{.}$ Here each $Y_{\text{ijh}}$ is a categorical variable, with possible values $r=1,\ldots ,R_{h}\text{.}$ Let $Z_{\text{ij}}={\left(Z_{i}^{H\prime },Z_{\text{ij}}^{L\prime }\right)}^{\prime }$ be a vector of observed covariates, where the variables in $Z_{\text{ij}}^{L}$ (*lower-level covariates*) can vary between different lower-level units within the same higher-level unit but $Z_{i}^{H}$ (*higher-level covariates*) vary only between the higher-level units. We take $Z_{i}^{H}$ to include a constant 1, thus introducing an intercept term to all the regression models described below.

The items $Y_{\text{ij}}$ are regarded as observed indicators of a discrete latent variable $X_{\text{ij}}$ with categories (*latent classes*) t=1,…,T. The standard latent class (LC) model specifies the joint probability function of $X_{\text{ij}}$ and $Y_{\text{ij}}$ as $P(Y_{\text{ij}},X_{\text{ij}})=P(X_{\text{ij}})P(Y_{\text{ij}}|X_{\text{ij}})\text{.}$ This has two basic elements, the *structural model* $P(X_{\text{ij}})$ for the probabilities of the latent classes, and the *measurement model* $P(Y_{\text{ij}}|X_{\text{ij}})$ for how the items measure the latent classes. We make throughout the assumption, which is standard in LC analysis, that $Y_{\text{ijh}}$ for different *h* are conditionally independent of each other given the latent class. The measurement model can then be written as

(1) $$P(Y_{\text{ij}}|X_{\text{ij}})=\prod_{h=1}^{H}P(Y_{\text{ijh}}|X_{\text{ij}}).$$

Next, the model is extended to accommodate the hierarchical structure of the data. This is done by expanding the structural model to $P(X_{\text{ij}},W_{i})=P(W_{i})P(X_{\text{ij}}|W_{i})\text{,}$ where $W_{i}$ is another categorical latent class variable, with categories m=1,…,M. It varies only between higher-level units *i*, so we refer to it as the *higher-level LC variable* and $X_{\text{ij}}$ as the *lower-level LC variable*. It is assumed that $Y_{\text{ij}}$ and $W_{i}$ are conditionally independent given $X_{\text{ij}}\text{,}$ and that $X_{\text{ij}}$ for the same *i* are conditionally independent given $W_{i}\text{.}$ Averaged over $P(W_{i})\text{,}$ however, values of $X_{\text{ij}}$ for different *j* within the same group *i* will be associated because they share the same $W_{i}\text{.}$ In this sense, $W_{i}$ is a categorical analogy of continuous random effects in multilevel (random effects) models, and the model is referred to as a *multilevel* (here two-level) *LC model*.

We then introduce covariates to the structural model, as

(2) $$P(X_{\text{ij}},W_{i}|Z_{\text{ij}})=P(W_{i}|Z_{i}^{H})P(X_{\text{ij}}|W_{i},Z_{\text{ij}}),$$

noting that higher-level classes $W_{i}$ can only depend on higher-level covariates $Z_{i}^{H}$ but lower-level classes $X_{\text{ij}}$ can depend on both lower- and higher-level covariates. We specify these models as the multinomial logistic models

(3) $$P(W_{i}=m|Z_{i}^{H})=\frac{\;\text{exp}\;(\alpha_{m}^{\prime }Z_{i}^{H})}{\sum_{l=1}^{M}\;\text{exp}\;(\alpha_{l}^{\prime }Z_{i}^{H})}\;\text{ and }$$

(4) $$P(X_{\text{ij}}=t|W_{i}=m,Z_{\text{ij}})=\frac{\;\text{exp}\;(\gamma_{t|m}^{\prime }Z_{\text{ij}})}{\sum_{s=1}^{T}\;\text{exp}\;(\gamma_{s|m}^{\prime }Z_{\text{ij}})},$$

where $\alpha_{m}$ and $\gamma_{t|m}$ for m=1,…,M and t=1,…,T are parameter vectors, and $\alpha_{1}=0$ and $\gamma_{1|m}=0$ for all *m* for identifiability. The specification may include constraints on the parameters, for example when some of them are 0 or when matching elements of $\gamma_{t|m}$ are equal for all *m*. Often the focus of substantive interest is on model (4) for the lower-level latent class $X_{\text{ij}}\text{,}$ and the higher-level class $W_{i}$ is regarded just as a random effect to allow for within-group associations between $X_{\text{ij}}\text{.}$ In that case, model (3) will often include just the intercept terms $\alpha_{m}=\alpha_{m}\text{.}$

The model defined by (1) and (2) is a standard multilevel LC model with covariates (Bakk et al., 2022; Di Mari et al., 2023; Lyrvall et al., 2024; Vermunt, 2003). A key feature of it is that the measurement model (1) does not depend on $Z_{\text{ij}}\text{.}$ This can be relaxed by introducing covariates also to this, as

$$P(Y_{\text{ij}}|X_{\text{ij}},Z_{\text{ij}})=\prod_{h=1}^{H}P(Y_{\text{ijh}}|X_{\text{ij}},Z_{\text{ijh}}^{*})$$

where the models for the individual items are multinomial logistic models

(5) $$P(Y_{\text{ijh}}=r|X_{\text{ij}}=t,Z_{\text{ijh}}^{*})=\frac{\;\text{exp}\;(\delta_{\text{hr}|t}^{\prime }Z_{\text{ijh}}^{*})}{\sum_{q=1}^{R_{h}}\;\text{exp}\;(\delta_{\text{hq}|t}^{\prime }Z_{\text{ijh}}^{*})}$$

for $r=1,\ldots ,R_{h}\text{,}$ and $\delta_{\text{hq}|t}$ are parameter vectors with $\delta_{h1|t}=0$ for all *h*, *t*. This kind of measurement model for item $Y_{h}$ is *non-equivalent* with respect to the covariates in $Z_{\text{ijh}}^{*}\text{.}$ We write this with the subscript *h* to denote only those elements of Z which do affect the measurement model for the *h*th item. This is useful for clarity, because it is very common that these include only a subset of the variables in Z, and that they are different for different items. There may be parameter constraints, for example so that the coefficients of $Z_{\text{ijh}}^{*}$ (except for the intercept) do not depend on latent class *t*, or that even for the same *h* they may be non-zero for some latent classes but zero for others. If $Z_{\text{ijh}}^{*}$ includes only the constant 1, measurement of item $Y_{\text{ijh}}$ is *equivalent* with respect to all of the covariates.

Let $Y_{i}={\left(Y_{i1}^{\prime },\ldots ,Y_{in_{i}}^{\prime }\right)}^{\prime }$ and $Z_{i}={\left(Z_{i1}^{\prime },\ldots ,Z_{in_{i}}^{\prime }\right)}^{\prime }$ denote all the observed values of the items and the covariates for higher-level unit *i*. The model for these observed data is obtained by averaging over the distributions of the latent $W_{i}$ and $X_{\text{ij}}\text{,}$ as

$$\begin{array}{c} P(Y_{i}|Z_{i};\theta )\\ =\sum_{m=1}^{M}(P(W_{j}=m|Z_{i}^{H};\theta_{2})\\ \times \prod_{j=1}^{n_{i}}\left\{\sum_{t=1}^{T}P(X_{\text{ij}}=t|W_{j}=m,Z_{\text{ij}};\theta_{2})\left[\prod_{h=1}^{H}P(Y_{\text{ijh}}|X_{\text{ij}}=t,Z_{\text{ijh}}^{*};\theta_{1})\right]\right\}) \end{array}$$

where we have also introduced parameters $\theta ={\left(\theta_{1}^{\prime },\theta_{2}^{\prime }\right)}^{\prime }$ into the notation. Here $\theta_{1}$ denotes all the parameters of the measurement model, i.e., the δ s in (5), and $\theta_{1}$ all the parameters of the structural model, i.e., the α s and γ s in (3) and (4).

Model (6) is a multilevel (here two-level) latent class model with covariates and with non-equivalence of measurement. What we examine in this paper is two-step methods of estimating the parameters of this model, with focus on the structural parameters $\theta_{2}\text{.}$ In the general presentation of the method in Section 3 we take the choice of $Z_{\text{ij}1}^{*},\ldots ,Z_{\text{ijH}}^{*}$ as given, i.e., we assume that it has already been determined which covariates are needed to allow for non-equivalence of measurement in different items. Model selection procedures for deciding on this are described by Masyn (2017) and Vermunt and Magidson (2021); an illustration of them is included in our applied example in Section 5. We also assume that the specification of the measurement model is such that the parameters of the structural model are formally and practically identified. This requires, in essence, that the non-equivalence should not be too extensive, at a minimum that it does not affect all of the items in $Y_{\text{ij}}\text{.}$

## 3. Two-Step Estimation of the Model Parameters

The $Y_{i}$ for different higher-level units *i* are taken to be conditionally independent given $Z_{i}\text{.}$ The log-likelihood function for the model that was defined in Section 2 can then be written as $l(\theta )=l(\theta_{1},\theta_{2})=\sum_{i=1}^{I}\;\text{log}\;P(Y_{i}|Z_{i};\theta )\text{,}$ where $P(Y_{i}|Z_{i};\theta )$ is given by (2) combined with (3)–(5).

One-step maximum likelihood (ML) estimates of the parameters are obtained by maximizing l(θ) with respect to all of θ at once. In contrast, two-step estimation divides the estimation into two steps. In its step 1, an estimate ${\tilde{\theta }}_{1}$ of the measurement parameters is obtained. In step 2, estimates ${\tilde{\theta }}_{2}$ of the structural parameters are obtained by maximizing $l({\tilde{\theta }}_{1},\theta_{2})$ with respect to $\theta_{2}\text{,}$ i.e., using the same log-likelihood as for one-step estimation but treating now the measurement parameters $\theta_{1}$ fixed at their estimated values ${\tilde{\theta }}_{1}$ from step 1.

This idea of two-step estimation has been examined for single-level latent class models by Bakk and Kuha (2018) and for multilevel LC models by Di Mari et al. (2023). What is new here is that we want to extend it to the case where the model includes non-equivalence of measurement. The key question is then how step 1 should be carried out. The general answer is that it should use the simplest model that allows valid estimation of $\theta_{1}\text{.}$ To present this, we write now $Z_{\text{ij}}={\left(Z_{\text{ij}}^{†\prime },Z_{\text{ij}}^{*\prime }\right)}^{\prime }\text{,}$ where $Z_{\text{ij}}^{*}$ denotes the union of $Z_{\text{ijh}}^{*}$ over *h*, i.e., those covariates that appear in the measurement model for at least one item, and $Z_{\text{ij}}^{†}$ denotes those covariates that do not appear anywhere in the measurement model. Let $p(Z_{\text{ij}}^{†}|Z_{\text{ij}}^{*})$ denote the conditional joint distribution of $Z_{\text{ij}}^{†}$ given $Z_{\text{ij}}^{*}\text{.}$ The conditional distribution for the latent class variables and the items given $Z_{\text{ij}}^{*}$ only is obtained by marginalising over this, as

(7) $$\begin{array}{c} \begin{array}{c} P(Y_{\text{ij}},X_{\text{ij}},W_{i}|Z_{\text{ij}}^{*};\theta_{1},\theta_{2}^{*}) \end{array}\\ \begin{array}{c} =\left[\int P(X_{\text{ij}},W_{i}|Z_{\text{ij}}^{†},Z_{\text{ij}}^{*};\theta_{2})p(Z_{\text{ij}}^{†}|Z_{\text{ij}}^{*})dZ_{\text{ij}}^{†}\right]\;P(Y_{\text{ij}}|X_{\text{ij}},Z_{\text{ij}}^{*};\theta_{1})\\ =P(X_{\text{ij}},W_{i}|Z_{\text{ij}}^{*};\theta_{2}^{*})P(Y_{\text{ij}}|X_{\text{ij}},Z_{\text{ij}}^{*};\theta_{1})\\ =P(W_{i}|Z_{i}^{H*};\theta_{2}^{*})P(X_{\text{ij}}|W_{i},Z_{\text{ij}}^{*};\theta_{2}^{*})P(Y_{\text{ij}}|X_{\text{ij}},Z_{\text{ij}}^{*};\theta_{1}). \end{array} \end{array}$$

This is of the same multilevel LC form as the full model given $Z_{\text{ij}}$ which led to (2). The two have different structural models, since (7) is conditional on $Z_{\text{ij}}^{*}$ only (so we denote its structural parameters by $\theta_{2}^{*}$ rather than $\theta_{2}$). Crucially, however, both have the same measurement model $P(Y_{\text{ij}}|X_{\text{ij}},Z_{\text{ij}}^{*};\theta_{1})\text{,}$ with the same $\theta_{1}\text{.}$ The measurement parameters $\theta_{1}$ can thus be estimated from this, using an observed-data log likelihood that is obtained by marginalising (7) over $X_{\text{ij}}$ and $W_{i}\text{,}$ This is the key result that was derived by Vermunt and Magidson (2021) for step 1 of three-step estimation for single-level LC models, and it holds also for two-step estimation for the multilevel models that we consider here. Vermunt and Magidson (2021) also observed that the same result holds even if the model includes observed variables that are treated as distal outcomes rather than covariates, even when they depend on the items $Y_{\text{ij}}\text{;}$ this is because they would be integrated out from an expression like (7). If the model has full measurement equivalence, i.e., $Z_{\text{ij}}^{*}$ includes only the constant 1, (7) integrates out all the covariates. The step-1 model is then a multilevel LC model without covariates, as in Di Mari et al. (2023).

We note that this derivation involves one approximation. This is that if the structural models given $Z_{\text{ij}}$ are multinomial logistic models as in (3) and (4), they will in general be only approximately of a multinomial logistic form given a smaller set $Z_{\text{ij}}^{*}$ (unless this is empty or includes only a single categorical variable). We do not expect that this will have a meaningful impact on the quality of the step-1 estimates of $\theta_{1}$ (we note also that the same inconsistency arises whenever any multinomial logistic models are fitted given different sets of covariates, even for observed response variables).

In summary, when there is non-equivalence of measurement with respect to covariates $Z_{\text{ij}}^{*}\text{,}$ step 1 of two-step estimation should be for a model which includes these $Z_{\text{ij}}^{*}$ in both the structural model and the measurement model. This is still simpler than one-step estimation if $Z_{\text{ij}}^{*}$ is smaller than the full set of covariates $Z_{\text{ij}}\text{.}$ Estimates ${\tilde{\theta }}_{1}$ of the measurement parameters from this step 1 are carried forward to step 2 (and estimates of the structural parameters $\theta_{2}^{*}$ are discarded). Two-step estimates ${\tilde{\theta }}_{2}$ of the structural parameters are then obtained from step 2 by maximizing $l({\tilde{\theta }}_{1},\theta_{2})$ with respect to $\theta_{2}\text{.}$

For estimation of standard errors of ${\tilde{\theta }}_{2}\text{,}$ two broad approaches are possible. One of them accounts for sampling uncertainty in ${\tilde{\theta }}_{1}$ by including a term corresponding to this in the standard error calculation (Bakk and Kuha 2018; Di Mari et al. 2023). The other, simpler approach, omits this term, in effect taking the estimated measurement model from step 1 as an a priori fixed definition of the latent classes (see Kuha and Bakk 2023 for a discussion of these options). In our applied example in Section 5 we use this simpler approach to calculate the standard errors.

## 4. Simulation Study

### 4.1. Design

We use a simulation study to examine the performance of the proposed two-step method of estimation for multilevel latent class models with measurement non-equivalence (abbreviated *MNE* below). We focus on results for estimated parameters of the structural model for the lower-level classes (model (4) in Section 2), because this is typically the focus of substantive research questions in applications of multilevel LC models. Our primary question of interest is how well the estimates perform when MNE is correctly specified in the measurement model, and a secondary question is how much bias they have when MNE is incorrectly ignored and equivalence of measurement is assumed. For both of these questions, we also use one-step estimation as a comparator.

Two main factors are varied in the simulation settings: separation of the latent classes (i.e., the strength of the measurement model) and magnitude of the MNE. It is well known for models without MNE that estimates behave better when the classes are more clearly separated (Bakk & Kuha, 2018; Di Mari et al., 2023; Lyrvall et al., 2024; Vermunt, 2010), and we would expect the same to be the case here. Similarly, we expect that estimation is more demanding if non-equivalence is more pronounced. A question of interest is then how large these differences may be.

Each simulated sample has I=100 higher-level units *i* and $n_{i}=100$ lower-level units *j* in each *i*. Each higher-level unit belongs to one of two known groups, identified by an observed variable $G_{i}=0,1\text{.}$ The value of $G_{i}$ is drawn at random for each *i*, with probability $P(G_{i}=1)=0.5\text{.}$ Non-equivalence of measurement may exist between these groups. This structure might correspond, for example, to a multicultural educational study where the higher-level units are schools, lower-level units are students, and the two groups are two different languages of instruction in the schools.

We consider models with T=3 lower-level latent classes (categories of $X_{\text{ij}}$) and M=2 higher-level latent classes (categories of $W_{i}$). Model (3) for $W_{i}$ has no covariates, i.e., $Z_{i}^{H}=1\text{,}$ and we set $P(W_{i}=1)=0.6$ and $P(W_{i}=2)=0.4\text{.}$ Model (4) for $X_{\text{ij}}$ has $G_{i}$ as its only covariate, i.e., $Z_{\text{ij}}={\left(1,G_{i}\right)}^{\prime }\text{.}$ The intercepts of this model are set so that, averaged over the distribution of $G_{i}\text{,}$ we have $P(X_{\text{ij}}=1|W_{i}=1)=P(X_{\text{ij}}=3|W_{i}=2)=0.18\text{,}$ $P(X_{\text{ij}}=2|W_{i}=1)=P(X_{\text{ij}}=2|W_{i}=2)=0.31\text{,}$ and $P(X_{\text{ij}}=3|W_{i}=1)=P(X_{\text{ij}}=1|W_{i}=2)=0.51\text{.}$

In all of the simulations, in the model for $X_{\text{ij}}$ all coefficients of $G_{i}$ (i.e., in all $\gamma_{t|m}$ in (4) for t=2,3 and m=1,2) are equal to 0.5. The estimated model correctly assumes that these coefficients do not vary by the higher-level class *m*, so that the model has two estimable coefficients of $G_{i}\text{.}$ These are the parameters we focus on, considering all of their estimates together.

The lower-level latent class is measured by H=6 binary items $Y_{\text{ijh}}$ for h=1,…,H, each with values 0 and 1. Consider the item response probabilities $\pi_{h(t)g}=P(Y_{\text{ijh}}=1|X_{\text{ij}}=t,G_{i}=g)\text{.}$ Here for simplicity we write $G_{i}$ in place of the covariates $Z_{\text{ijh}}^{*}$ because in all cases where there is MNE we have $Z_{\text{ijh}}^{*}=(1,G_{i})$ (and when there is no MNE, $Z_{\text{ijh}}^{*}=1$ and $\pi_{h(t)0}=\pi_{h(t)1}$). In all settings $\pi_{h(t)g}$ has a high value (>0.5) for all items h=1−6 in the first lower-level class (t=1), for items 1–3 in class t=2 and for no items in class t=3, and low probabilities (≤0.5) otherwise. In different simulations we then allow MNE by group $G_{i}$ for some of the $\pi_{h(t)g}\text{.}$ The strength of class separation and magnitude of MNE are determined by how these probabilities vary and how far they are from 0.5.

We consider simulation conditions with weaker and stronger lower-level class separation separately for low and high values of $\pi_{h(t)g}\text{,}$ resulting in four settings for class separation. These are combined with three conditions for MNE—none, weak and strong—resulting in 12 simulation conditions in total. When there is MNE, it affects the measurement models of some items in latent classes 1 and 2 but none of them in class 3. In the weaker MNE condition, classes 1 and 2 have MNE for items h=1,2. In the stronger condition, class 1 has MNE in items 1–4 and class 3 in items 1–3. Thus MNE here affects only those probabilities $\pi_{h(t)g}$ that are greater than 0.5. In each case its effect is to shift the response probability down by 0.1 for group 1, i.e., $\pi_{h(t)1}=\pi_{h(t)0}-0.1\text{.}$ The resulting values of the response probabilities in the twelve simulation conditions are summarised in Table 1.

**Table 1.** Values of the item response probabilities in different conditions considered in the simulations.

Patterns of response probabilities:							
		Response probability					
		$\pi_{h(t)g}=P(Y_{h}=1|X=t,G=g)$					
		for item (*h*)					
Class (*t*)	Group (*g*)	1	2	3	4	5	6
1	0	$H_{0a}$	$H_{0a}$	$H_{0b}$	$H_{0b}$	*H*	*H*
	1	$H_{1a}$	$H_{1a}$	$H_{1b}$	$H_{1b}$	*H*	*H*
2	0	$H_{0a}$	$H_{0a}$	$H_{0b}$	*L*	*L*	*L*
	1	$H_{1a}$	$H_{1a}$	$H_{1b}$	*L*	*L*	*L*
3	0	*L*	*L*	*L*	*L*	*L*	*L*
	1	*L*	*L*	*L*	*L*	*L*	*L*

Values of the probabilities in different simulation conditions:							
	Separation	Separation	Measurement				
Condition	(low $\pi_{h(t)g}$)	(high $\pi_{h(t)g}$)	non-equiv.	($H_{0a},H_{1a}$)	($H_{0b},H_{1b}$)	*H*	*L*
1	Weak	Weak	None	0.8	0.8	0.8	0.5
2	Weak	Strong	None	0.9	0.9	0.9	0.5
3	Strong	Weak	None	0.8	0.8	0.8	0.2
4	Strong	Strong	None	0.9	0.9	0.9	0.1
5	Weak	Weak	Weak	(0.8, 0.7)	0.8	0.8	0.5
6	Weak	Strong	Weak	(0.9, 0.8)	0.9	0.9	0.5
7	Strong	Weak	Weak	(0.8, 0.7)	0.8	0.8	0.2
8	Strong	Strong	Weak	(0.9, 0.8)	0.9	0.9	0.1
9	Weak	Weak	Strong	(0.8, 0.7)	(0.8, 0.7)	0.8	0.5
10	Weak	Strong	Strong	(0.9, 0.8)	(0.9, 0.8)	0.9	0.5
11	Strong	Weak	Strong	(0.8, 0.7)	(0.8, 0.7)	0.8	0.2
12	Strong	Strong	Strong	(0.9, 0.8)	(0.9, 0.8)	0.9	0.1

In the lower table, two values for $\left(H_{0a},H_{1a}\right)$ and/or $\left(H_{0b},H_{1b}\right)$ indicate that the values of these probabilities are different in groups g=0,1, i.e., that there is measurement non-equivalence in the corresponding part of the model.

For each of the conditions, we generate 250 random samples. The data analysis is carried out in Mplus (Muthén & Muthén, 2017) and R (R Core Team, 2024), using the package MplusAutomation (Hallquist & Wiley, 2018).

### 4.2. Results

Tables 2 and 3 show the simulation results, in the form of the average bias, root mean squared error (RMSE) and median absolute error (MAE) of estimates over the 250 simulations in each of the simulation scenarios. As noted above, the parameters considered here are the two coefficients of $G_{i}$ in the model for the lower-level class $X_{\text{ij}}\text{,}$ both with the true value of 0.5. We consider their estimates together, so that we have 500 estimated values for each simulation setting.

**Table 2.** Estimation assuming full equivalence of measurement.

Class separation for		True level of measurement non-equivalence					
		None		Weak		Strong	
(low $\pi_{h(t)g}$)	(high $\pi_{h(t)g}$)	One-step	Two-step	One-step	Two-step	One-step	Two-step
Mean bias:							
Weak	Weak	0.001	−0.012	0.263	0.055	0.743	0.256
Weak	Strong	0.003	−0.002	0.031	0.073	0.153	0.258
Strong	Weak	−0.003	−0.003	0.028	0.027	0.128	0.126
Strong	Strong	0.000	−0.001	0.009	0.008	0.058	0.057
Root mean squared error:							
Weak	Weak	0.125	0.121	1.238	0.412	1.888	0.741
Weak	Strong	0.088	0.086	0.312	0.267	0.520	0.480
Strong	Weak	0.067	0.067	0.137	0.135	0.225	0.218
Strong	Strong	0.058	0.058	0.079	0.079	0.118	0.116
Median absolute error:							
Weak	Weak	0.084	0.080	0.533	0.361	0.930	0.683
Weak	Strong	0.055	0.057	0.288	0.244	0.514	0.438
Strong	Weak	0.048	0.048	0.116	0.114	0.181	0.168
Strong	Strong	0.036	0.036	0.059	0.059	0.090	0.089

Mean bias, root mean squared error (RMSE) and median absolute error (MAE) of two-step and one-step estimates of the structural parameters. The results are across the 2×250 estimates of two coefficients of the covariate *G* (both with true value of 0.5) in the model for lower-level latent class *X*, over 250 simulation replications in each of the twelve simulation conditions in Table 1.

**Table 3.** Estimation under correctly specified model for measurement non-equivalence (MNE).

Class separation for		Level of measurement non-equivalence			
		Weak		Strong	
(low $\pi_{h(t)g}$)	(high $\pi_{h(t)g}$)	One-step	Two-step	One-step	Two-step
Mean bias:					
Weak	Weak	0.002	−0.021	−0.066	−0.118
Weak	Strong	0.003	−0.011	−0.008	−0.101
Strong	Weak	−0.003	−0.006	−0.002	−0.056
Strong	Strong	−0.001	−0.001	0.004	−0.007
Root mean squared error:					
Weak	Weak	0.159	0.127	0.560	0.187
Weak	Strong	0.098	0.086	0.222	0.134
Strong	Weak	0.072	0.066	0.103	0.087
Strong	Strong	0.059	0.058	0.060	0.054
Median absolute error:					
Weak	Weak	0.101	0.086	0.287	0.124
Weak	Strong	0.069	0.059	0.139	0.109
Strong	Weak	0.049	0.045	0.067	0.064
Strong	Strong	0.037	0.035	0.041	0.035

Mean bias, root mean squared error (RMSE) and median absolute error (MAE) of two-step and one-step estimates of the structural parameters. The results are across the 2×250 estimates of two coefficients of the covariate *G* (both with true value of 0.5) in the structural model for lower-level latent class *X*, over 250 simulation replications in each of the eight simulation conditions in table 1 that involve MNE.

Consider first estimation where measurement non-equivalence is ignored, i.e., when both two-step and one-step estimates are calculated under the assumption of full equivalence of measurement. These results are shown in Table 2. When the true model has no MNE, there is little difference between the two estimators and both are essentially unbiased. Both of them become increasingly seriously biased when the true measurement model involves increasing levels of MNE. This bias is also larger when class separation is weaker, i.e., when the measurement model is weak. Here there are also noticeable differences between the two estimators, in that the two-step estimates have mostly smaller bias and smaller RMSE than the one-step estimates, especially in the more difficult low-separation settings. The same is true for MAEs, showing that the poorer performance of the one-step estimates is fairly general and not just due to a small number of extreme values of the estimates.

Table 3 shows the results in the eight simulation conditions where MNE is present, when the estimators are based on a correct specification for the MNE. Both estimators again perform better when the separation between the latent classes is stronger. This is as expected, and consistent with previous results for estimation in situations with no MNE (e.g., Bakk & Kuha, 2018; Vermunt, 2010). Here the most challenging conditions are the ones where low item response probabilities (i.e., the ones indicated by ‘L’ in Table 1) are 0.5, so that they are not very clearly distinguished from the higher response probabilities. The estimators perform reasonably well, and in most cases essentially similarly. However, some differences between them emerge when class separation is weak and there is a large amount of MNE. Here the two-step estimates have a little more bias, but clearly lower RMSE and MAE than the one-step estimates. In these most difficult situations a large proportion of the one-step estimates are thus quite far from the true parameters, whereas two-step estimation substantially reduce these extremes.

## 5. Empirical Example

We illustrate the proposed two-step method of estimation for multilevel LC models with non-equivalence of measurement with an analysis of cross-national data on citizenship norms among adolescents. The data come from the International Civic and Citizenship Education Study 2016 (Schulz et al., 2018), which was conducted by the International Association for the Evaluation of Educational Achievement, and are accessed via the R package multilevLCA (Lyrvall et al., 2023). These data have been used in previous substantive studies of citizenship norms (Hooghe et al., 2016; Hooghe & Oser, 2015; Oser et al., 2023; Oser & Hooghe, 2013). For details on data cleaning and recoding, see [reference with DOI to be added].

The survey asked 14-year-old adolescents to state their level of agreement on whether a set of activities are important for a person to be considered a good adult citizen. We include responses to five such questions, related to activities that correspond to engaged citizenship: participation in local activities (we label this item *local*), engagement in political conversations (*discuss*), show of support for environmental protection activities (*envir*), promotion of human rights (*rights*), and participation in peaceful protests (*protest*). The responses are coded in a binary form, as 1 if the respondent regarded the activity as very or quite important for being a good adult citizen, and 0 if they thought it not very or not at all important.

These five binary variables are the measurement items ($Y_{\text{ijh}}$ in the notation above). Individual-level latent classes ($X_{\text{ij}}$) measured by them will characterise different profiles of what an adolescent considers important in a good citizen. We have hierarchical data where individual children (lower-level units *j*) are nested within countries (higher-level units *i*). We consider structural models where the proportions of $X_{\text{ij}}$ may vary by two country characteristics (non-constant covariates in $Z_{\text{ij}}=Z_{i}$), the country’s wealth and its civic freedom, specifically press freedom. We do not include covariates for the higher-level latent classes $W_{i}\text{,}$ so $Z_{i}^{H}$ in the notation of Section 2 includes only a constant. Wealth is measured by logarithm of gross domestic product in U.S. dollars (covariate *lnGDPusd*), and a covariate on press freedom is based on the 2016 World Press Freedom Index (PFI) by Reporters Without Borders. Civic freedom has previously not been considered as an explanatory variable in the latent class analysis citizenship norms literature. For clarity of this illustrative example, we consider data from two groups of countries which have very different levels of press freedom. Five of the countries are among those with the highest levels of PFI—Finland (ranked 1st), Netherlands (2), Norway (3), Denmark (4), and Sweden (8)—and three among the lowest—Colombia (134), Russia (148), and Mexico (149). We define a dummy variable (*lowPFI*) which is 1 for the countries in the low-PFI group and 0 for the high-PFI group.^1^ The sample sizes range from 2,728 (Netherlands) to 7,138 (Russia), with a total combined sample of 40,837 respondents.

We also allow for the possibility of MNE in some of the items, with respect to *lowPFI*. The two groups of countries defined by it have very different constraints on political expression, and the different activities mentioned in the survey items may have different relative salience for adolescents’ perceptions on what it takes to be a good citizen. In particular, we speculate that this may be the case for support for environmental protection, promotion of human rights, and participation in peaceful protests, which are more public and/or politically contentious activities. We therefore consider the possibility of MNE in these items. The citizenship norms literature has not previously analyzed civic freedom as a potential confounding variable in the identification of latent classes.

We first identified the optimal number of latent classes. This was based on the Bayesian information criterion (BIC) combined with considerations of substantive clarity of the estimated LC structure. A general recommendation is to perform this first step of model selection without covariates and under the assumption of equivalence of measurement (Masyn, 2017). We first estimated single-level models with one to five latent classes, and concluded that the four-class specification was preferred. We then estimated two-level LC models, with individual countries as the higher-level units, still with equivalence of measurement and without covariates. With four lower-level classes, the best BIC value was obtained for a model with three higher-level classes. This multilevel model is preferred to the four-class single-level model, indicating that allowing for the hierarchical clustering structure is desirable. We select the two-level model with four high-level and three low-level classes for the rest of the modeling.

In the second step of model selection, we add MNE with respect to *lowPFI* to this multilevel model. We consider it for all combinations of the three items *envir*, *rights*, and *protest*, both when allowing MNE to vary across classes and when restricting MNE to be invariant (on the logit scale) across classes (i.e., constraining the coefficient of *lowPFI* in $\delta_{\text{hr}|t}=\delta_{\text{hr}}$ in Equation (5) not to depend on latent class *t*). Here *lowPFI*, i.e., the covariate in $Z_{\text{ij}}^{*}\text{,}$ is included also in the model for the latent class variable $X_{\text{ij}}\text{.}$ The best BIC value is obtained for a model which includes class-invariant MNE in two items, *envir* and *protest*. In particular, it is preferred to a model with full equivalence of measurement. This indicates that MNE is present in the data.

Estimates of the measurement model parameters $\theta_{1}$ for the selected model from this step are also the step-1 estimates of these parameters for two-step estimation, as discussed in Section 3. The item response probabilities implied by this model are shown in Table 4. The first class places importance on all five items. The second class emphasizes the items related to specific topics (*local*, *envir*, *rights*), but not as much or at all the ones related to method of engagement (*discuss*, *protest*). Individuals belonging to the third class have middling probabilities of endorsing each of the items, and those in the fourth class do not place importance on any of them as criteria for a good adult citizen. We label class 1 *Maximal*, class 2 *Topic*, class 3 *Medium*, and class 4 *Unengaged*. The same interpretation of the classes would also be obtained from a model which constrains the measurement models to be fully equivalent, item probabilities from which are also shown in Table 4 for comparison. The implications for allowing for MNE are seen in the probabilities for items *envir* and *protest* in the selected model. Here in all classes the probabilities of endorsing these items are higher in countries with low press freedom. In other words, adolescents in countries with low levels of press freedom are more uniformly likely to regard support for environmental protection and participation in peaceful protests as characteristics of a good adult citizen than are adolescents in countries with more press freedom.

**Table 4.** Item response probabilities for the four lower-level (individual-level) classes, describing different profiles of engaged citizenship norms.

					Model with full			
	Selected model				measurement equivalence			
	Cl. 1	Cl. 2	Cl. 3	Cl. 4	Cl. 1	Cl. 2	Cl. 3	Cl. 4
Item	*Maximal*	*Topic*	*Medium*	*Uneng.*	*Maximal*	*Topic*	*Medium*	*Uneng.*
*local*	0.981	0.961	0.600	0.096	0.980	0.961	0.660	0.113
*discuss*	0.953	0.000	0.294	0.074	0.981	0.001	0.298	0.091
*rights*	0.988	0.988	0.668	0.000	0.985	0.981	0.730	0.032
*envir*					0.984	1.000	0.772	0.210
*lowPFI* =0	0.977	0.998	0.693	0.166				
*lowPFI* =1	0.987	0.999	0.796	0.255				
*protest*					0.879	0.668	0.360	0.065
*lowPFI* =0	0.831	0.562	0.330	0.035				
*lowPFI* =1	0.885	0.666	0.434	0.054				

The probabilities are shown for a model where the measurement models of items *envir* and *protest* are non-equivalent with respect to the binary covariate *lowPFI* (countries with high vs. low levels of press freedom), and for a model where all the measurement probabilities are equivalent across countries.

Table 5 shows the estimated proportions of the latent classes after this first step, again for the selected model and for the full equivalence model for comparison. For the selected model, these probabilities are averaged over the sample proportions of the two values of *lowPFI*. In broad terms, the most noticeable difference between the higher-level classes is that one of them (class 2 in the table) has substantially higher probabilities than the other two classes of individuals belonging to the two lower-level classes (class *Medium* and *Unengaged*) which place least importance on these items as indicators of good citizenship. Averaged over the probabilities of the higher-level classes, the estimated proportions of individuals in the lower-level classes in the selected model are 0.37, 0.26, 0.29 and 0.07 for the *Maximal*, *Topic*, *Medium* and *Unengaged* classes respectively.

**Table 5.** Estimated proportions of the three higher-level (country-level) latent classes, and of the four lower-level (individual-level) latent classes within the higher-level classes.

				Model with full		
	Selected model			measurement equivalence		
	Higher-level class (proportion)			Higher-level class (proportion)		
Lower-level class	1 (0.500)	2 (0.375)	3 (0.125)	1 (0.500)	2 (0.375)	3 (0.125)
*Maximal*	0.467	0.290	0.236	0.428	0.189	0.289
*Topic*	0.251	0.190	0.543	0.233	0.151	0.549
*Medium*	0.226	0.408	0.185	0.273	0.493	0.141
*Unengaged*	0.055	0.112	0.036	0.066	0.167	0.021

The probabilities are shown for the selected model where the measurement models of items *envir* and *protest* are non-equivalent with respect to the binary covariate *lowPFI* (and averaging over the sample distribution of this variable) and for a model where all the measurement probabilities are equivalent across countries.

Finally, we estimate the structural model for the individual-level latent class given the covariates *lowPFI* and *lnGDPusd*. The estimated coefficients of this multinomial logistic model are reported in Table 6, again showing results based on the selected measurement model with MNE and, for comparison, a model with full measurement equivalence. Table 6 shows two-step estimates of the parameters of the structural model, estimated as described in Section 3, and with the measurement parameters fixed at their estimated values from Table 4. The reference category for a respondent is here the class *Unengaged*. Considering the estimates from the selected model, the results show that adolescents living in countries with less press freedom are increasingly more likely to have norms that emphasize more activities, relative to having “unengaged” norms, even after controlling for GDP. The coefficients of *lnGDPusd* indicate that individuals in higher-GDP countries are most likely to belong to the class *Maximal* which regards all the activities as important for good citizenship, but less likely to belong to the class *Topic* which de-emphasises the role of discussion only and (to a lesser extent) participation in peaceful protests. These differences between the more engaged classes (*Maximal* and *Topic*) and the less engaged classes (*Medium* and *Unengaged*) are substantively large and statistically significant (by conventional criteria) with respect to both covariates. In contrast, neither covariate makes a significant difference on the distinction between the two less engaged classes.

**Table 6.** Estimated coefficients of the covariates *lowPFI* (dummy variable for countries that have low press freedom Index) and *lnGDPusd* (country’s log GDP in US dollars) in a multilevel model for individual-level latent classes.

	Coefficient (in model vs. class *Unengaged*)					
				Model with full		
	Selected model			measurement equivalence		
Covariate	*Maximal*	*Topic*	*Medium*	*Maximal*	*Topic*	*Medium*
*lowPFI*	0.963***	1.657***	0.423	1.125***	1.987***	0.619***
*lnGDPusd*	0.561***	$-0.371^{**}$	0.184	0.516*	$-0.440^{*}$	0.059

*p < 0.05 ,

**p < 0.01 ,

***p < 0.0001 .

These estimates are from the second step of two-step estimation. The measurement model for the items given the latent classes is fixed at the estimated parameters of the selected model which allows for measurement non-equivalence in two items (on the left) or of a model where all the measurement probabilities are equivalent across countries (on the right). The fixed measurement probabilities of these two choices are as shown in Table 4.

Comparing the estimates under the two specifications on measurement, we can see, in particular, that the coefficients of *lowPFI* are consistently less strong when we allow for measurement non-equivalence. This happens because some of the association between *lowPFI* and the responses is accounted for by measurement differences, that is, by the fact that the specific activities that have MNE are overall relatively more salient for adolescents in countries with low press freedom. where *lowPFI* is 1. Even after accounting for this, however, it is clear that adolescents in countries with low levels of press freedom are substantially more likely to be of the view that good citizenship is something that encompasses a larger number of activities.

## 6. Concluding Remarks

We proposed a two-step estimation approach for multilevel latent class models with covariates in the presence of measurement non-equivalence. The method involves estimating the measurement model in the first step, and then holding its parameters fixed at their estimated values in the second step where the structural model for the classes given covariates is estimated. The key modification that is needed here, compared to two-step estimation of models with full measurement equivalence, is that covariates which create non-equivalence of measurement need to be included already in the first step. Their direct effects on measurement indicators are estimated there, while their coefficients (and those of any other covariates) in the structural model are estimated in the second step.

From a simulation study we observed that the proposed estimator performs generally well when the model is correctly specified, and essentially as well as the one-step maximum likelihood estimator which estimates all parameters at once. The performance of both estimators deteriorates to some extent in settings where the measurement model is weak and there is strong non-equivalence of measurement. The simulations also gave some evidence that two-step estimates are more robust to model misspecification which occurs when the measurement is incorrectly taken to be equivalent.

We have argued that two-step estimation has in principle two kinds of advantages over one-step estimation, the computational and the conceptual. The conceptual one is that estimating and fixing the measurement model before we proceed to estimate structural models for the latent classes means that the *definition* of the classes is then also fixed, and will not change even if we estimate and compare multiple different structural models. This advantage holds unchanged even when the models involve measurement non-equivalence. The computational advantage of two-step estimation, on the other hand, is somewhat reduced here. This is because the first step now includes also those covariates that are needed to account for the non-equivalence, making this step too more complex in comparison to when there is an absence of non-equivalence. It remains the case, however, that thereafter estimation is less demanding than it would be in one-step estimation. This is because in the two-step approach it will involve only the structural parameters, whereas the measurement parameters are fixed rather than repeatedly re-estimated.

As always, some questions on the properties and procedures of these methods are left open. We mention in particular questions of model selection. For multilevel latent class models with measurement non-equivalence this involves multiple dimensions: choosing the number of latent classes at the lower and higher levels, as well as determining which covariates are involved in non-equivalence and in what ways. Decisions on these dimensions could affect each other. In this paper we did not examine this question but employed a particular approach in line with previous literature. However, more systematic understanding of different approaches that could be used here would still be desirable.

## Footnotes

1 An alternative analytical approach would be to use the original continuous PFI score, which is ranging from 0 to 100. In this empirical example, we focus on the binary *low* -*high* classification for ease of interpretation. Because the variation in PFI score between the five countries is substantially larger between these two groups than within these groups, we expect this choice of analytical approach has little qualitative impact on the results (among the low-PFI countries, the PFI scores are 55.89, 50.97, and 50.67 for Colombia, Russia, and Mexico, respectively; among the high-PFI countries, they are 91.41, 91.24, 91.21, 91.11, and 87.67 for Finland, Netherlands, Norway, Denmark, and Sweden, respectively).
